# Assessing the Impacts of Local Knowledge and Technology on Climate Change Vulnerability in Remote Communities

**DOI:** 10.3390/ijerph8030733

**Published:** 2011-03-04

**Authors:** Christopher Bone, Lilian Alessa, Mark Altaweel, Andrew Kliskey, Richard Lammers

**Affiliations:** 1 Resilience and Adaptive Management Group, University of Alaska Anchorage, 3101 Science Circle, Anchorage, AK 99508, USA; E-Mails: Christopher.Bone@nrcan.gc.ca (C.B.); afadk@uaa.alaska.edu (A.K.); 2 Computation Institute, University of Chicago, 5735 South Ellis Avenue, Chicago, IL 60637, USA; E-Mail: maltaweel@anl.gov; 3 Institute for the Study of Earth, Oceans, and Space, University of New Hampshire, Durham, NH 03824, USA; E-Mail: Richard.Lammers@unh.edu

**Keywords:** vulnerability, climate change, technology-induced environmental distancing, traditional ecological knowledge, agent-based modeling

## Abstract

The introduction of new technologies into small remote communities can alter how individuals acquire knowledge about their surrounding environment. This is especially true when technologies that satisfy basic needs, such as freshwater use, create a distance (*i.e.*, diminishing exposure) between individuals and their environment. However, such distancing can potentially be countered by the transfer of local knowledge between community members and from one generation to the next. The objective of this study is to simulate by way of agent-based modeling the tensions between technology-induced distancing and local knowledge that are exerted on community vulnerability to climate change. A model is developed that simulates how a collection of individual perceptions about changes to climatic-related variables manifest into community perceptions, how perceptions are influenced by the movement away from traditional resource use, and how the transmission of knowledge mitigates the potentially adverse effects of technology-induced distancing. The model is implemented utilizing climate and social data for two remote communities located on the Seward Peninsula in western Alaska. The agent-based model simulates a set of scenarios that depict different ways in which these communities may potentially engage with their natural resources, utilize knowledge transfer, and develop perceptions of how the local climate is different from previous years. A loosely-coupled pan-arctic climate model simulates changes monthly changes to climatic variables. The discrepancy between the perceptions derived from the agent-based model and the projections simulated by the climate model represent community vulnerability. The results demonstrate how demographics, the communication of knowledge and the types of ‘knowledge-providers’ influence community perception about changes to their local climate.

## 1. Introduction

Community perceptions of climate change are constructed by the relationships that individuals share with their environment, the nature in which communities are structured, and the rate at which climate variables change over time [1]. Perceptions play a crucial role in the ability of a community to adapt to climate change as misguided views can impede a group’s response or ability to cope with external stresses, leaving them vulnerable [2]. The relationship between community perceptions and vulnerability is particularly important when addressing the needs of marginalized communities. Communities with relatively small populations that exist in remote locations often lack sufficient resources and infrastructure to adapt to stresses such as changes in temperature and precipitation regimes that affect their traditional way of life [3]. Furthermore, community vulnerability is exacerbated when community perceptions about their environment are adversely affected by the introduction of new technologies that alter the way in which they access traditional resources [4]. Traditional methods for accessing freshwater, such as collecting freshwater from natural local water sources, are altered when municipal water systems (*i.e.*, indoor piping or water delivery) are introduced [5]. The change in practice may provide greater convenience to a community and potentially increase their health, but it fosters a distancing between individuals and their environment as they no longer have to engage in the act of water collection, which in turn diminishes their experiences with their surrounding landscape. As a result, this process, referred to as *technology-induced environmental distancing* (TIED) [5], adversely impacts the ability of a community to adapt to climate change because their environmental perceptions become compromised.

In contrast to the process of TIED, local knowledge (LK) regarding one’s surrounding environment reinforces a knowledgebase of the environment that has been developed over many years by individuals in a community. LK represents a cumulative body of knowledge, practices and beliefs of human-environment relationships existing within a community [6,7]. There exist multiple concepts describing the construct of knowledge within a community and the transmission of knowledge between generations, such as traditional environmental knowledge, traditional knowledge and indigenous knowledge [8]. While these concepts share numerous similarities, we use the term *local knowledge* in this study to describe an accumulated knowledge base in a community over time regarding local climatic and hydrological systems. Local knowledge has become an important source of information for detecting local impacts from climate change [9–15], in addition to providing information for environmental monitoring [16,17], sustainable agriculture practices [18,19], natural resource use [20–22] and land conservation [23].

While it is clear that TIED and LK impose conflicting tensions on community vulnerability, it remains uncertain how these two processes interact with each other to influence individuals’ perceptions of their local climate, and how this leads to the emergence of an overall community perception that drives local decision-making. In order to gain perspective on the tension between TIED and LK, the objective of this study is to develop an agent-based modeling approach for simulating how community perceptions evolve over time when subject to changes in technology and local knowledge, and how these dynamic perceptions influence the vulnerability of a community to climate change. An agent-based model (ABM) is developed in which community members are represented by individual agents who perceive the current state of the environment and compare it to their knowledgebase of the past. Their perceptions, which can be influenced by LK, are amalgamated to form an emergent community perception. The nature in which the emergent perception is formed is dependent on community demographics and agent types; that is, agents influence their community’s perception of the environment based on their age and their willingness to engage in the well-being of the community. The outcome of the model is a time series of measurements of community vulnerability to climate change, which, for the purpose of this study, is defined as the discrepancy between perceived and recorded indicators of climate change.

The model is applied to two remote communities in western Alaska who are currently experiencing adverse climate change impacts and whose environmental perceptions are influenced by TIED due to the increase reliance on municipal water systems rather than traditional forms of non-municipal water collection. Previous research has shown that a conversion to municipal water systems from non-municipal water systems creates a distancing effect in remote communities that influences the perceptions of individuals about their environment [24]. A set of scenarios are simulated that represent varying relationships between TIED and LK that these communities can potentially experience in order to assess how the complex interactions amongst community structure, technology and local knowledge affect a community’s vulnerability to climate change. The simulation results for the set of scenarios are used to address four questions:

1. How do community demographic dynamics impact community perceptions of climate change?
2. How does the conversion from traditional resource use to non-traditional resource use influence community perceptions of climate change?
3. How does the inclusion of local knowledge influence community perceptions and mitigate adverse impacts of TIED on community perceptions?
4. How is community perception influenced by community structure?

A description of the modeling of agents, community demographic dynamics and traditional resource use behaviour is provided below.

## 2. Methods

**Figure 1.**
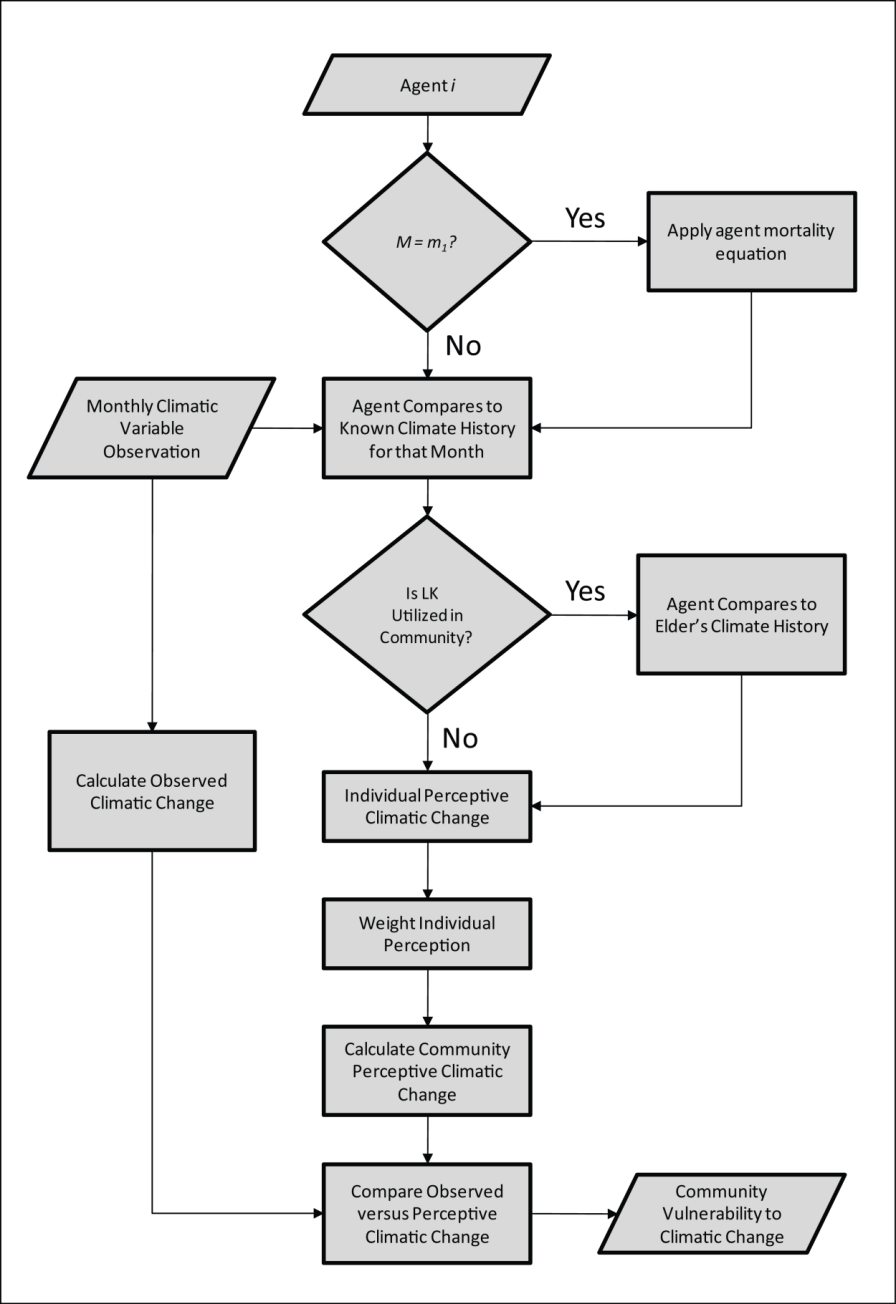
The overall flow of the agent-based model.

The objective of the model is to understand how community perceptions about climate change emerge from quantitative perceptions of individuals in the community, their interactions with each other, the influence that technology imposes on their perceptions, and the transmission of local knowledge from one generation to another. All parameters used in the model are described in Table 1 and are discussed in detail in the following sub-sections, and the flow of the model is illustrated in Figure 1.

### 2.1. Agents

Each individual is represented by a single agent that possesses the ability to observe the current state of the environment at time steps representing a single month and compare it to the average state of the environment from the past, pass its local environmental knowledge onto the community, receive local environmental knowledge from older community members, and make decisions regarding the use of non-municipal versus municipal freshwater resources. Each agent is defined by the following set of variables: age, age class, proportion of time engaged in a form of traditional resource use (TRU), *t_TRU_*, agent type, individual perception weight, *w_p_*, and knowledge transfer weight, *w_k_* (each variable is described below).

Agents are assigned to one of three age classes that are based on the previous work of Altaweel *et al.* [25]; these classes demonstrated distinguishable difference in freshwater resource use. Agents between 18–39 years of age fall into the *younger individuals* (*Y*) age class; agents 40–59 fall into the *middle-aged individuals* (*M*) age class; those agents over 60 years of age belong to the *older individuals* (*O*) age class. The age class structure is used to define the variable *t_TRU_* for the different simulated scenarios. For example, a specific scenario can be constructed in which *t_TRU_* for the *O* and *M* age classes are held constant while *t_TRU_* for the *Y* age class is diminished in order to simulate the vulnerability of communities over time when the youth of the population is continually impacted by the consequences of TIED.

Agent type classification in this research was derived from Alessa and Kliskey [26] while Altaweel *et al.* [25] provides a means for classifying individuals based on the nature in which they make decisions regarding the use of resources. Individuals in the community are considered either alpha (α), beta (β) or gamma (γ) agents. Alpha agents are initiators that attempt to promote and sustain efforts towards minimizing community vulnerability. Beta agents differ slightly in that they are concerned with the overall wellbeing of the community, but they do not initiate action to address community vulnerability. Gamma agents are primarily self-serving that can be persuaded to agree with movements towards minimizing community vulnerability but require some form of perceived self-benefit in order to do so. A further background on defining agent types can be found in the agent-based modeling literature [27–29]. A typology approach is useful in the context of this study because it facilitates the grouping of individuals into classes in which freshwater use is more common amongst class members than it is with members of other classes. Furthermore, it allows for the development of a weighting schematic (described below) that represents the influence of certain agent types in the overall perception of the community.

The concept of agent types was utilized in this study in order to establish the influence that individual agents impose on a community’s perception of climate change and the manner in which local knowledge is transferred between agents. Knowledge acquired from previous research [25,26] suggests that alpha agents have the greatest influence on community perception, followed by beta agents then gamma agents. Similarly, *O* agents exude greater influence than *M* agents, who both have greater influence than *Y* agents. This knowledge was used for defining a weighting scheme to define *w_p_*—a real number in the interval [0,1] expressing the influence that an individual agent has on community perception (Table 2). The weighting values were derived by establishing a proportional influence that can be parameterized as a real number between 0 and 1.

The probability of agent *i* becoming deceased at each time step of the model depends on the age of the eldest individual in the community. In the context of this research, establishing community demographics is often challenging as there is a sufficient lack of data existing over time. Therefore, the age of the eldest individual in the community provides a general estimate of life expectancy. In this regard, an agent in a community with greater life expectancy will have a lower probability of mortality compared to an agent in a community with a lower life expectancy. This assumption is implemented using the age of the oldest individual as the denominator in the equation. The probability of mortality is calculated as

(1) $$m=\frac{i_{\text{age}}^{e}}{\text{max}{\left[i_{{\text{age}}_{1}},\;i_{{\text{age}}_{2}}\;\ldots i_{{\text{age}}_{n}}\right]}^{e}}$$

where the inclusion of *e* indicates that mortality is based on an exponential function. The mortality equation is implemented once every twelve months.

The model developed in this study assumes that community population remains constant over time. While, in the context of this research, population levels do change over time, the process of population change is in itself a complex process that is not well understood in remote communities. Furthermore, while population levels have the potential of influencing community perception, the objective of the model is focused on the influence of community age and social structure.

The age structure of a community shifts as agents become deceased and new agents enter. When an agent becomes deceased, it is replaced by a *Y* agent with a *t_TRU_* that is equivalent to the average of the *t_TRU_* is in the *Y* age class. This ensures that the behaviour of new agents is influenced by existing agents of approximately the same age. Furthermore, the new agent takes on the agent type of the deceased agent to represent the potential of the new agent deriving from the family of the deceased and acquiring its behavioural traits.

With regards to the transfer of local knowledge, Table 3 presents the weighting scheme to define *w_k_*, a real number in the interval [0,1] that represents the amount of knowledge that is passed between individuals of different types. The weights in the table explain that full knowledge is passed between two agents when both are alphas, and the amount of transfer diminishes when involving beta agents, minimal knowledge is transferred when the provider of knowledge is a gamma agent, and no knowledge is transferred when the recipient is a gamma agent.

The next level in the modeling hierarchy is the community, which exhibits an overall perception of climate change based on the collection of individual perceptions. Community perception is compared to the actual change that has occurred in the environmental variables, and the difference between the two is considered to be the amount of vulnerability exhibited by the community. The highest level in the modeling hierarchy is the environment, represented by individual variables such as temperature and precipitation that are observable by the agents and therefore represents a one-way flow of information from the environment to the agents. Such variables may not represent the resource with which agents are interacting, but instead may impact the resource directly or indirectly, and are observable when agents are engaged with traditional resource use.

### 2.2. Estimating Community Vulnerability

At each time step of the model, an agent determines the difference between the current state of the environment and the state of the environment from the past. The agent first observes environmental variable *x* from the current month (*i.e.*, time step) and subtracts the average of environmental variable *x* for that month from its known history. An agent’s known history is determined by its age, *i_age_* and the amount of time it engages in traditional resource use. That is, variable *x* for month *m* in year *y_p_* exists in agent *i*’s known history if *i_age_* < *y_c_* *– y_p_*, and *t_TRU_* > δ, where *y_c_* and *y_p_* are the current year and a previous year, respectively, and δ is a real number in the interval [0,1] drawn from a random uniform distribution. This ensures that agents who engage more with traditional resource use have a more complete history of environmental variables. Agent *i*’s perception of change in variable *x* is thus estimated as

(2) $$p_{i,x}=x_{i,m,y_{c}}-\left[\frac{\sum x_{i,m,y_{p}}}{n}\right]$$

where *x_m,yp_* is the value of the environmental variable for a given year *y_c_* that exists in the agent’s knowledgebase.

In order to improve its knowledge of the local environment, an agent can access the environmental perceptions of another agent. However, an agent will only seek to acquire knowledge from another agent if the latter has historical climate knowledge that encompasses a longer time period. To determine the agent from which to acquire knowledge, agent *i* looks at the community population and, for all other agents, *j*, calculates

(3) $$i_{LK}=\text{max}\left[\frac{i_{\text{age}}}{\text{max}\left[i_{\text{age}}\right]}\times t_{TRU}\right]$$

This ensures that the agent selected to provide LK possesses experience about the environment as defined by both age and the amount of time engaged in traditional resource use.

Change in environmental variable *x* is calculated as

(4) $$q_{x}=x_{m,y_{c}}-\left[\frac{\sum x_{m,y_{p}}}{n}\right]$$

which explains that change is measured as the difference between the current observation of variable *x* and the average of variable *x* that is calculated over time. Community perception is calculated as the weighted sum of each agent`s individual perception. It is calculated using the equation

(5) $$P_{c,x}=\sum \left(\frac{w_{i}}{\sum w_{i}}p_{i,x}\right)$$

where *w_i_* is derived from Table 2. This equation explains that community perception is the sum of weighted perceptions of each individual in the community.

Community vulnerability *v* to changes in variable *x* is calculated as the difference between actual and perceived climate change using the equation

(6) $$v_{x}=q_{x}-P_{c,x}$$

As a result, higher values of *v*_x_ indicate a community whose perception of climate change is different than what is actually occurring.

## 3. Implementation

### 3.1. Study Sites and Social Data

**Figure 2.**
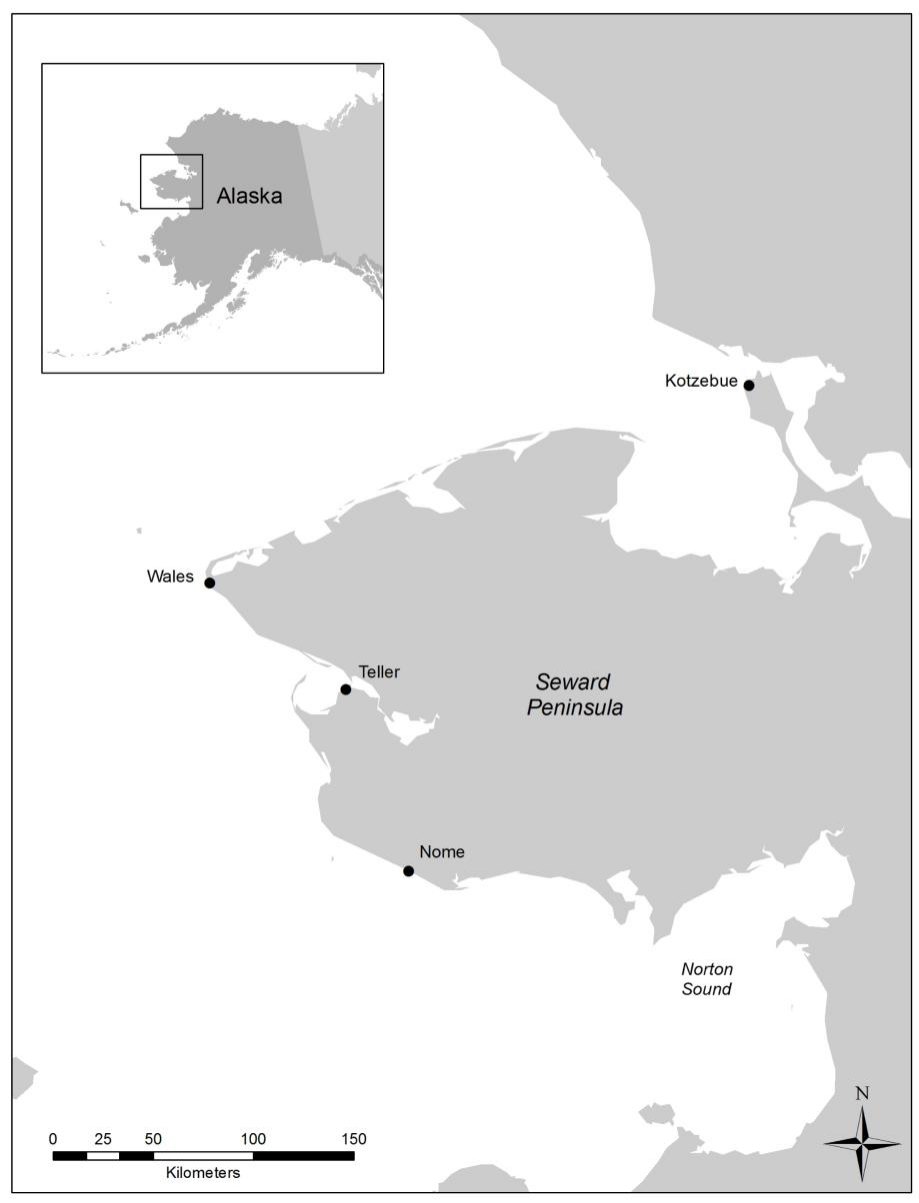
The study sites of Wales and Teller in western Alaska.

**Figure 3.**
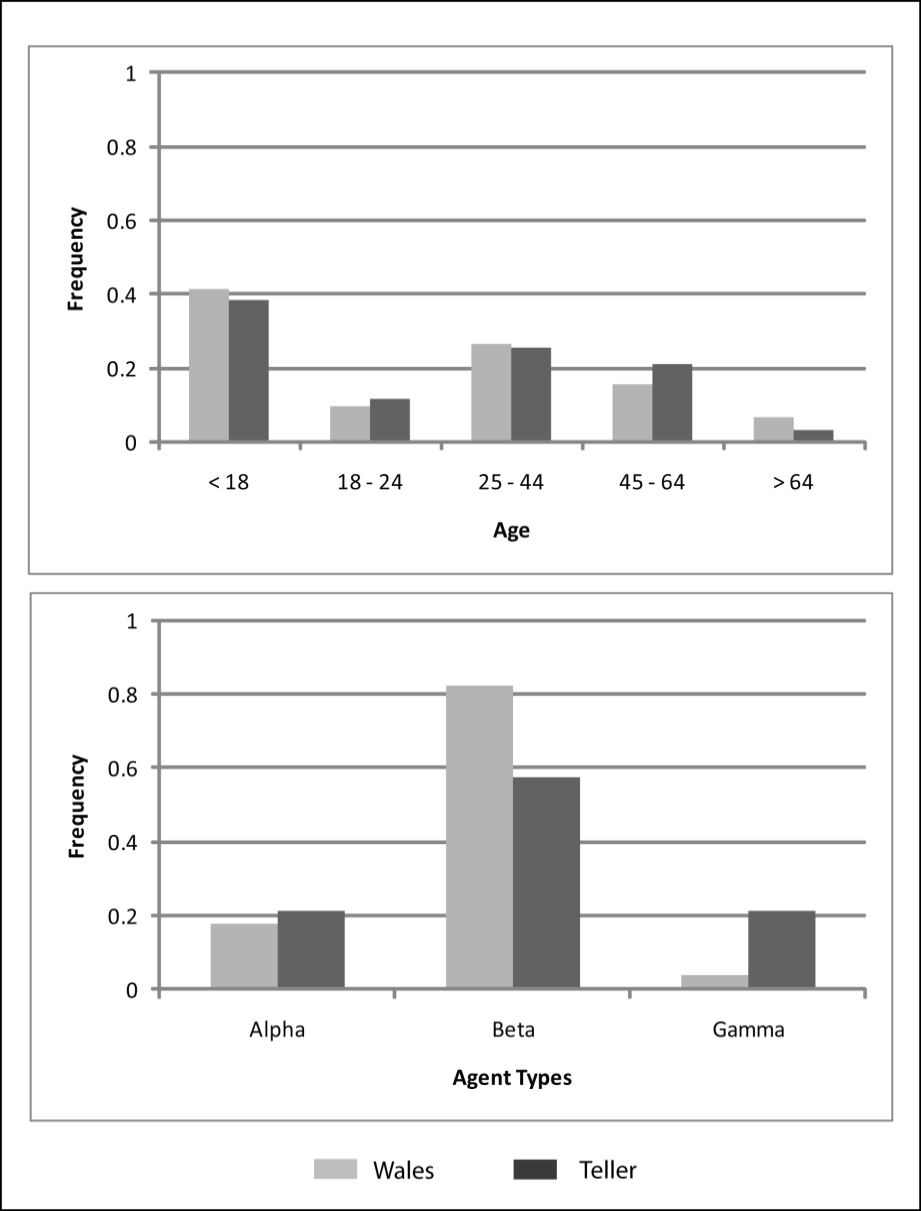
Community structure of Wales and Teller showing normalized frequencies by agent age and agent type classes.

The model was implemented for two remote communities in rural Alaska in order to simulate how their relationship with freshwater resources impacts their vulnerability to climate change. Specifically, the communities of Wales and Teller (Figure 2) were used to demonstrate how a shift from non-municipal water systems (NMS), such as rivers, creeks, shallow lakes and water tanks, to municipal water systems (MWS), such as piped or bottled water, influences a community’s ability to accurately perceive changes to the environment. Temperature, precipitation and runoff were selected as the three environmental variables that agents analyze in order to observe climate change. According to the 2000 U.S. census, the communities of Wales and Teller have populations of approximately 152 and 269, respectively, and with relatively similar demographics. Previous in situ fieldwork [4,30] provided data describing the social structure of these communities that facilitated the classification of individuals into agent types [25]. The age class structure and agent type distribution of the sample from each community is shown in Figure 3. The minimum age of eighteen was used in this model as previous research demonstrated that at this age agents become engaged in the decisions surrounding the use of freshwater resources. The two study sites demonstrate similar demographic structures but differences in the proportion of individuals belonging to the different agent type categories.

### 3.2. Environmental Data

Temperature and precipitation data covering 141 years from 1960–2100 were generated from the ECHAM5 Global Climate Model using the 20C3M scenario for years in the 20th Century and the A1B scenario for years in the 21st Century. The data were collected as part of the World Climate Research Programme’s (WCRP) Coupled Model Intercomparison Project phase 3 (CMIP3) multi-model dataset [31] and obtained from the Program for Climate Model Diagnosis and Intercomparison [32]. These data are the same as those used for the Intergovernmental Panel on Climate Change Fourth Assessment Report [33]. The temperature data represent mean monthly air temperature, while precipitation is the total monthly precipitation, both of which were calculated for spatial grid cells covering a 25 km × 25 km area. The time series was bias corrected using observed gridded fields from [34] and projected onto the 25 km × 25 km Northern Hemisphere EASE-Grid [35].

The University of New Hampshire Water Balance Model (UNH-WBM; [36]) was driven by these data to provide local runoff in each grid cell. The UNH-WBM is a macro-scale hydrological model used to calculate components of the hydrological cycle under changing climate conditions. It is a grid-based, spatially distributed watershed model that predicts spatially and temporally-varying hydrologic variables operating over large domains. The model includes spatially and temporally varying predictions of runoff/discharge volumes, land surface evapotranspiration losses to the atmosphere, freeze-thaw dynamics (active layer depth) via a degree-day approach, and snowmelt runoff.

The datasets were joined to create a single long-term gridded time series covering the pan-Arctic. The latitude and longitude coordinates from the two study sites were used to identify the grid cells from which to extract the monthly temperature, precipitation and local runoff data for the full time span.

### 3.3. Modeling Scenarios

Six different scenarios were constructed with different degrees of NMS usage, and each was simulated in the presence and absence of LK (for a total of twelve scenarios). These scenarios were established in order to represent different potential relationships between individuals and the use of freshwater resources. These scenarios do not necessarily represent how the communities currently interact with freshwater, but instead provide a spectrum of possible behaviours that can help to inform how different types of actions influence community vulnerability to climate change. The six NMS scenarios are defined by the NMS of each age class as depicted in Table 4. A brief description is provided here of each scenario and the agent-freshwater relationship that is depicted:

- *Scenario with Perfect knowledge*: Each agent has perfect knowledge of the past environment from the age of eighteen. That is, the agents are able to accurately estimate how environmental variables have changed over time. This scenario provides a means to gain insight into how the model operates in an ideal case, and provides a benchmark of agent perceptions to which other scenarios can be compared.
- *Traditional resource use by all agents*: Each agent has imperfect knowledge of the past environment, but there is an extremely high level of interaction with NMS. This scenario represents a community that is able to maintain its traditional methods for sustaining their livelihood.
- *Diminishing NMS by younger agents*: The youngest agents in the community convert from NMS to MWS rather quickly over time, while middle-aged agents convert gradually. This represents a community in which older members attempt to sustain traditional resource use while younger generations are altering their behaviours due to modern technology.
- *Diminishing NMS by older agents*: The oldest agents in the community convert quickly to MWS and middle-aged agents convert more slowly. However, the youngest agents in the community retain their use of traditional water resources. This represents a community in which the introduction of technology is mostly aimed at older individuals while the youngest generation struggles to maintain traditional values.
- *Gradual diminishing of NMS by all agents*: All agents gradually convert from NMS to MWS, but the rate at which they convert is dependent on age.
- *Rapid diminishing of NMS by all agents*: All agents quickly convert from NMS to MWS, but, as with Scenario D, the rate at which they convert is dependent on age.

The model is simulated to represent a period between 2010 and 2090; however, the agents utilize the climate dataset dating back to 1960 to construct their perceptions at each time step. The dates used in this model are dependent on the availability of data and do not reflect the actual time period of knowledge available in communities. However, the use of these dates provides a means to determine how agent perceptions change over time and influence community vulnerability.

The ABM was run for a total of thirty simulations in order to account for the random effects that were encoded in the model to influence agent perceptions; the results are the average of the set of simulations. The community vulnerability results are presented for both study sites with and without LK for each scenario. The results are discussed with reference to the four questions posed in the introduction.

## 4. Results

**Figure 4.**
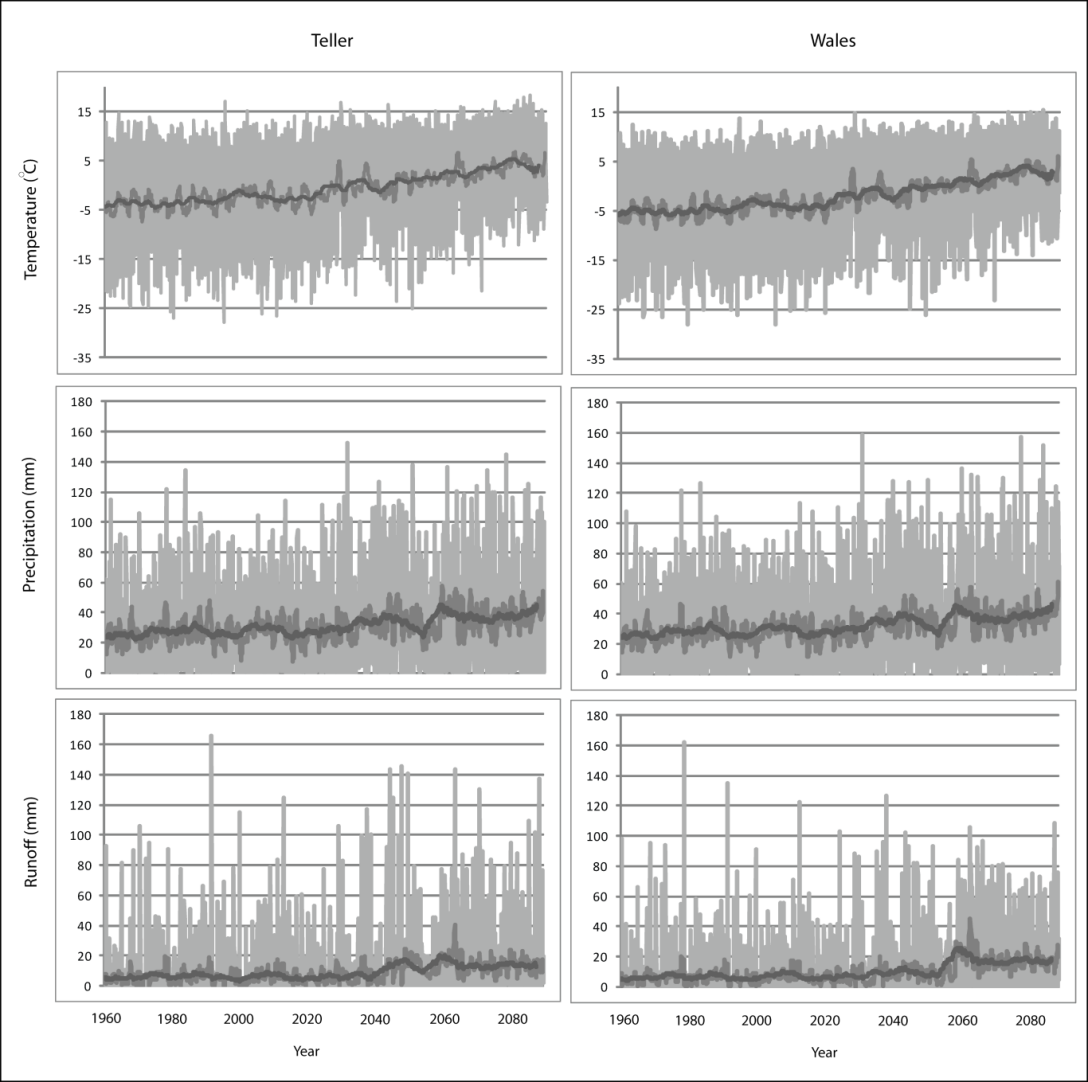
Temperature, precipitation and runoff from 1960 to 2090 derived from the climate and water balance model. Monthly (light grey), annual (dark grey) and the 5-year running mean (black line) show the upward trend of each variable.

Figure 4 illustrates monthly temperature, precipitation and runoff at Wales and Teller for the period between 1960 and 2090 showing the typical inter-annual variability of these time series for this part of Alaska. The graphs demonstrate an increase in all three variables. This observation is reiterated in Table 5, which shows the 5-year average of each variable from the start and end of the time series, and the difference between these two dates.

The graphs predict a relatively significant increase in all three variables. This observation is reiterated in Table 5, which shows the 5-year average of each variable from the start and end of the time series, and the difference between these two dates.

### 4.1. How Do Community Demographic Dynamics Impact Community Perceptions of Climate Change?

**Figure 5.**
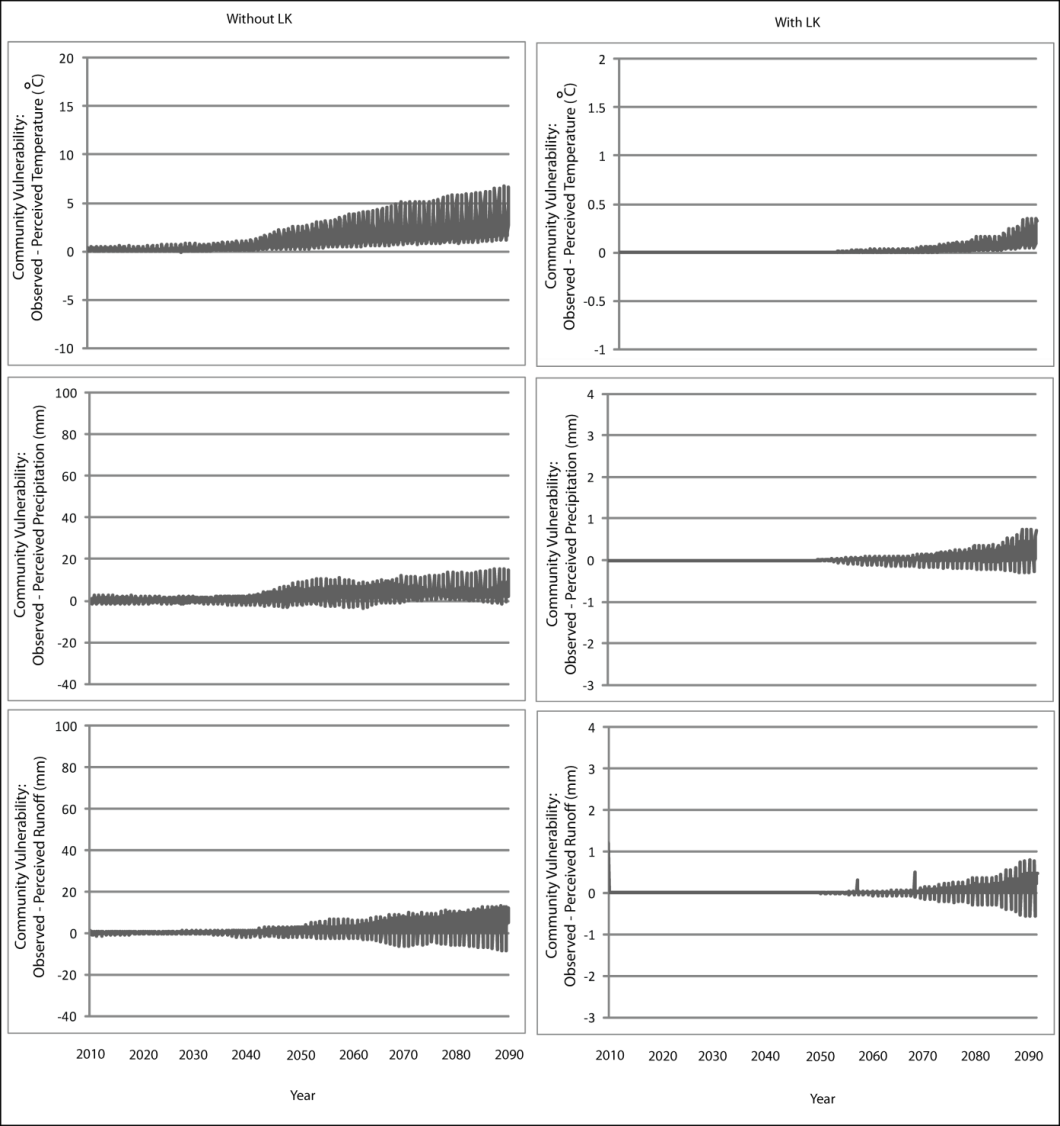
Simulation results from Scenario A depicting vulnerability to changes in temperature, precipitation and runoff for the communities of Wales with and without the inclusion of knowledge transmission.

**Figure 6.**
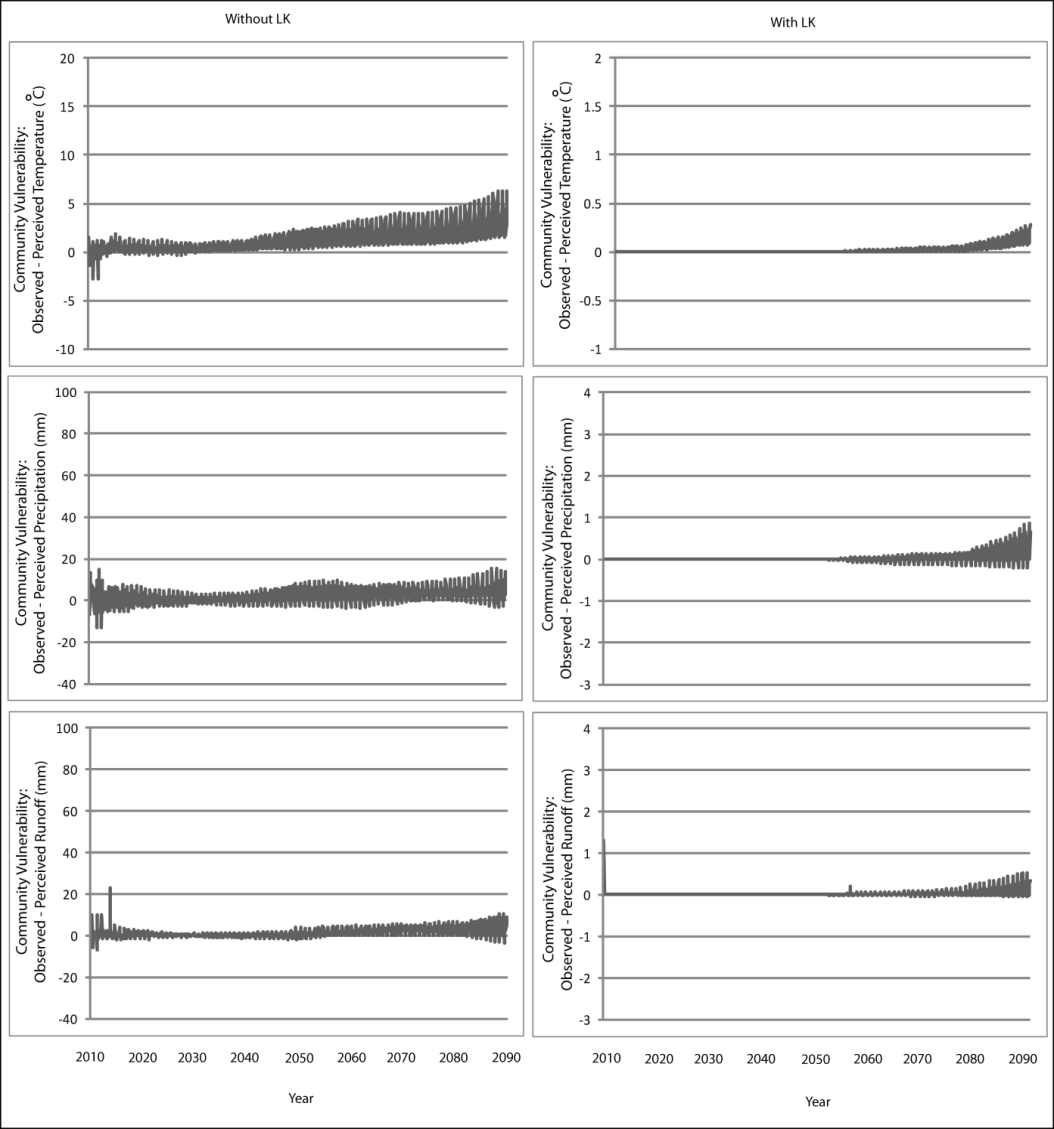
Simulation results from Scenario A depicting vulnerability to changes in temperature, precipitation and runoff for the communities of Teller with and without the inclusion of knowledge transmission.

The results from Scenario A with perfect agent knowledge are presented in Figures 5 and 6 for Wales and Teller, respectively. It is important to note here that the y-axis on the graphs depicting community vulnerability without LK are presented on a different scale than that with LK; this was done in order to adequately visualize the variation that exists with the vulnerability trajectories. The graphs demonstrate how demographic changes lead to increases in community vulnerability over time. In the scenario without knowledge transmission, older agents die and their knowledge about the past local climate is lost from community perceptions. Vulnerability continues to increase over time because temperature, precipitation and runoff are all increasing and becoming significantly different from those values in the initial years of the simulation—a time from which there is no longer existing knowledge about the local climate. By the end of the simulation, the difference between community perception and what is actually observed is almost equivalent to the actual observation itself. When this occurs, communities become at risk of having perceptions about climate change that are no better than if the perception was randomly generated. Demographics also play a role when knowledge transmission is integrated into community perceptions, although to a much less degree, the reasons for which are discussed below in Section 4.3.

### 4.2. *How Does the Conversion from Traditional Resource Use (*i.e., *NMS) to Non-traditional Resource Use (*i.e., *MWS) Influence Community Perceptions of Climate Change?*

**Figure 7.**
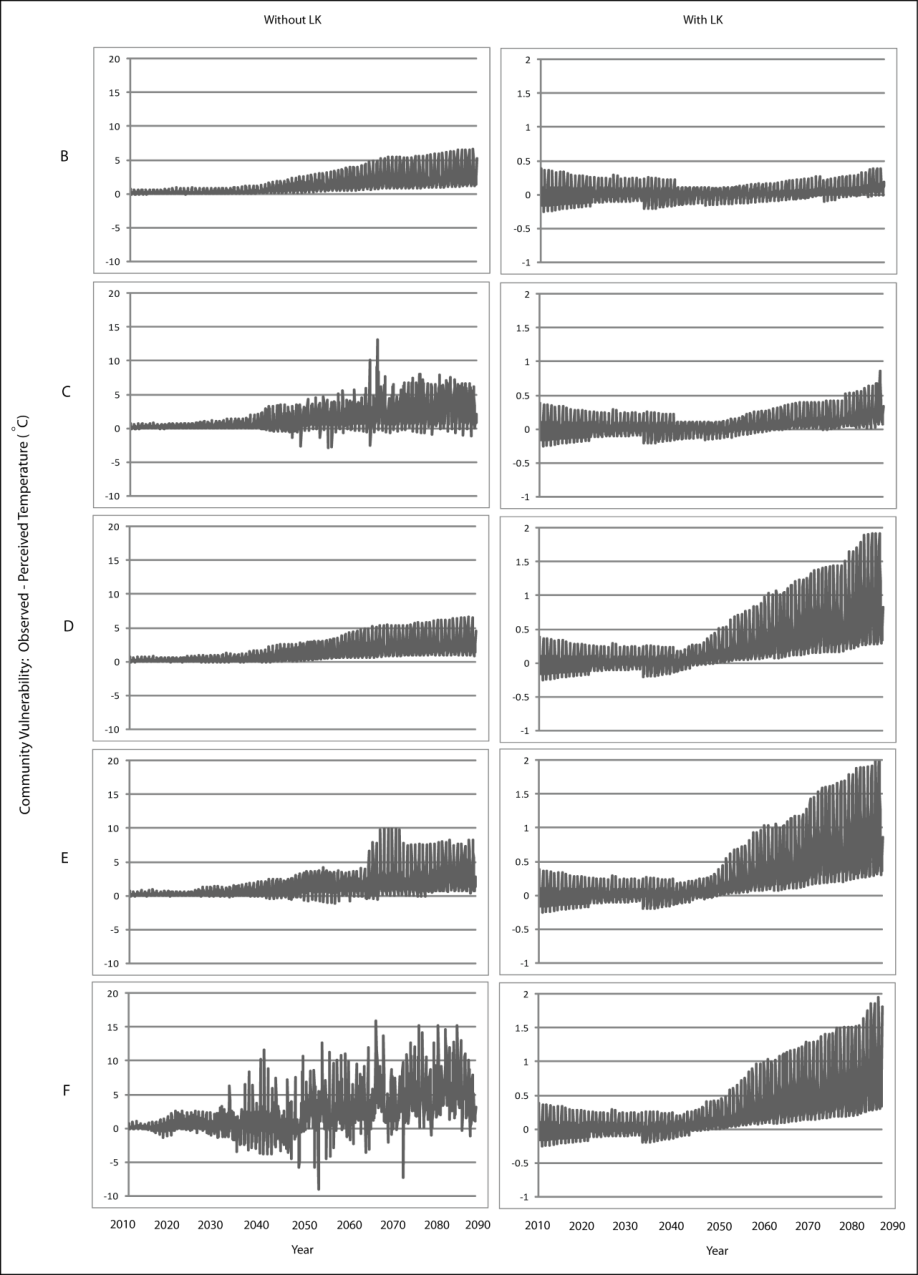
Simulation results from Scenarios B-F depicting vulnerability to temperature for Wales.

**Figure 8.**
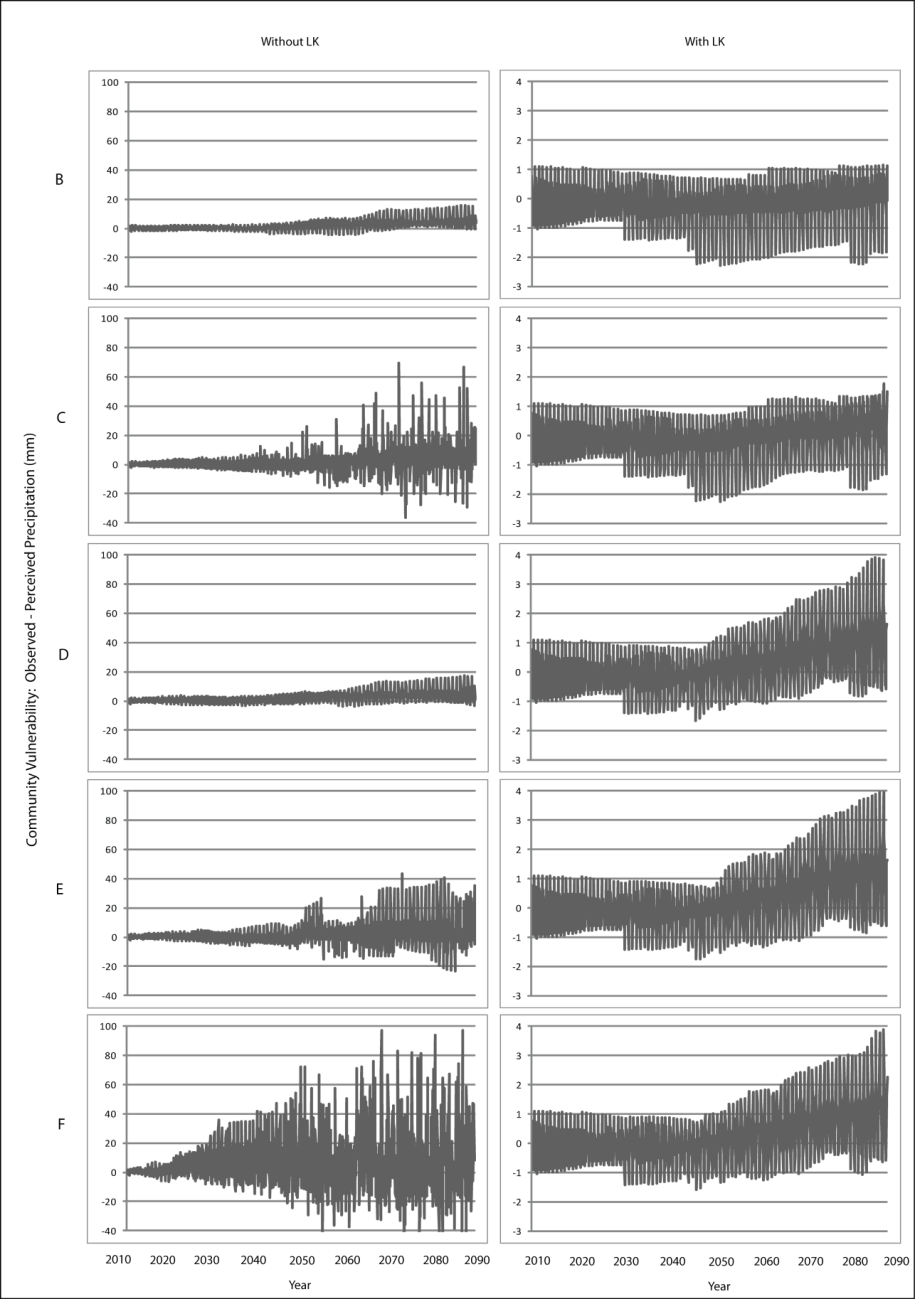
Simulation results from Scenarios B-F depicting vulnerability to precipitation for Wales.

**Figure 9.**
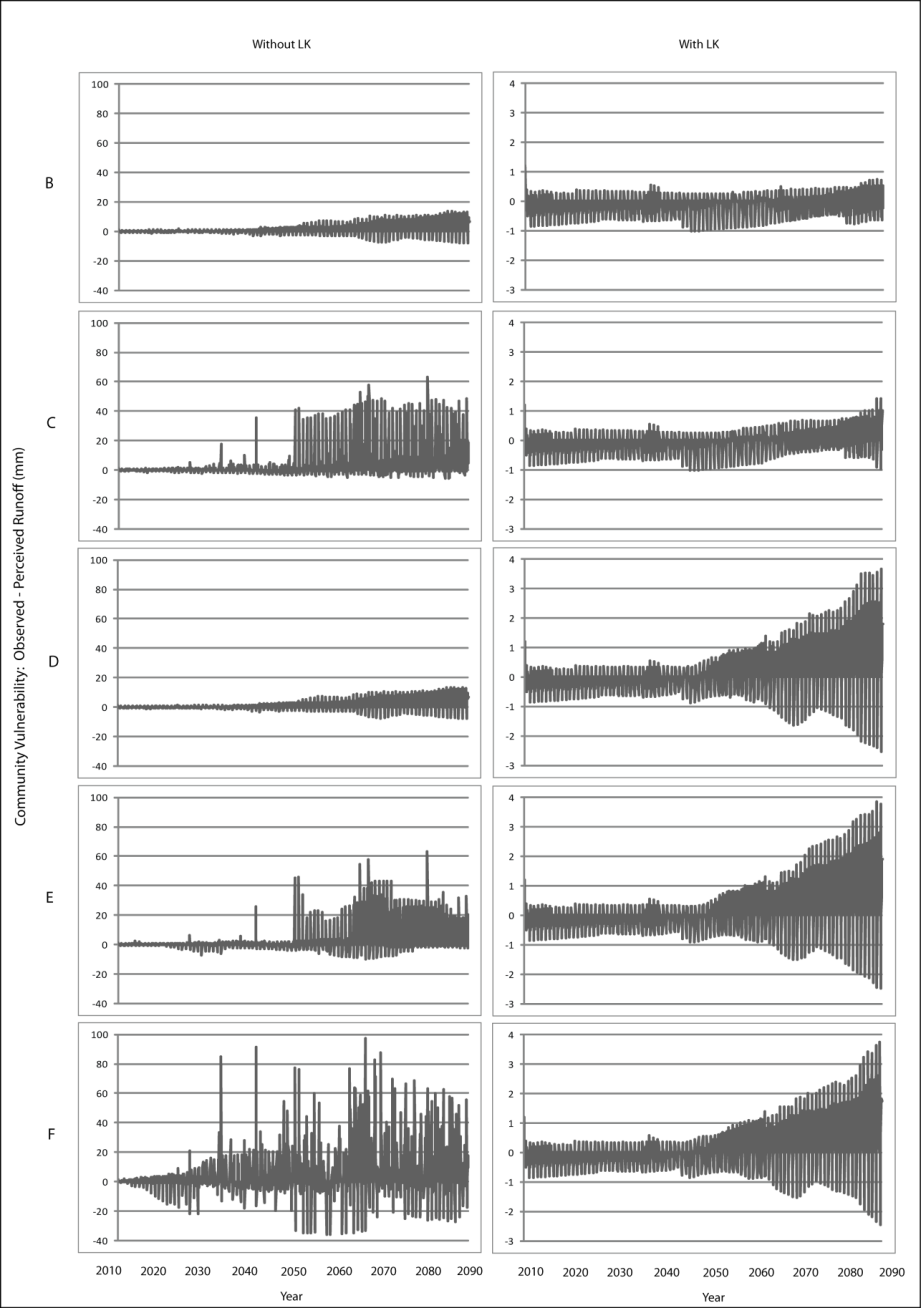
Simulation results from Scenarios B-F depicting vulnerability to runoff for Wales.

**Figure 10.**
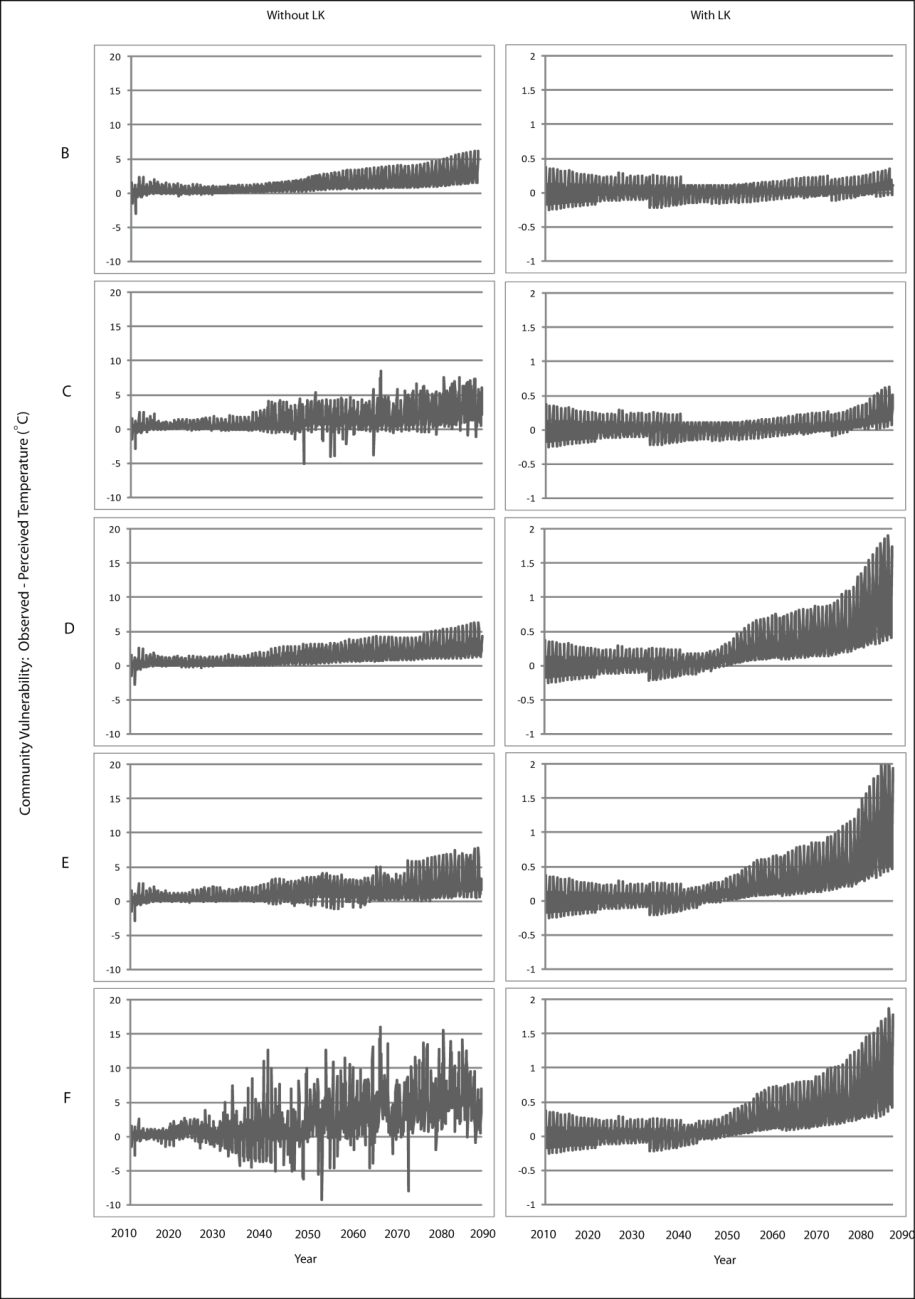
Simulation results from Scenarios B-F depicting vulnerability to temperature for Teller.

**Figure 11.**
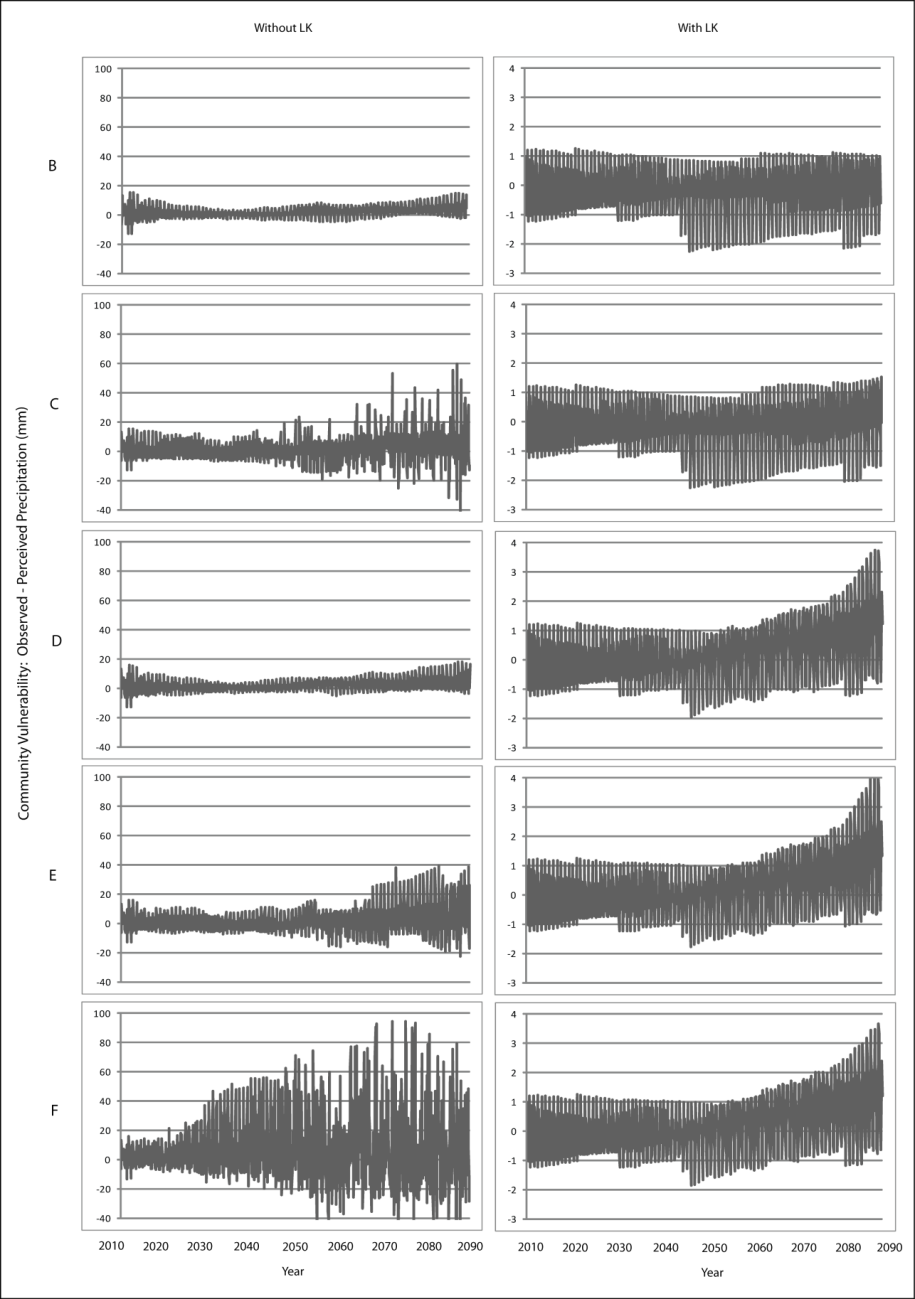
Simulation results from Scenarios B-F depicting vulnerability to precipitation for Teller.

**Figure 12.**
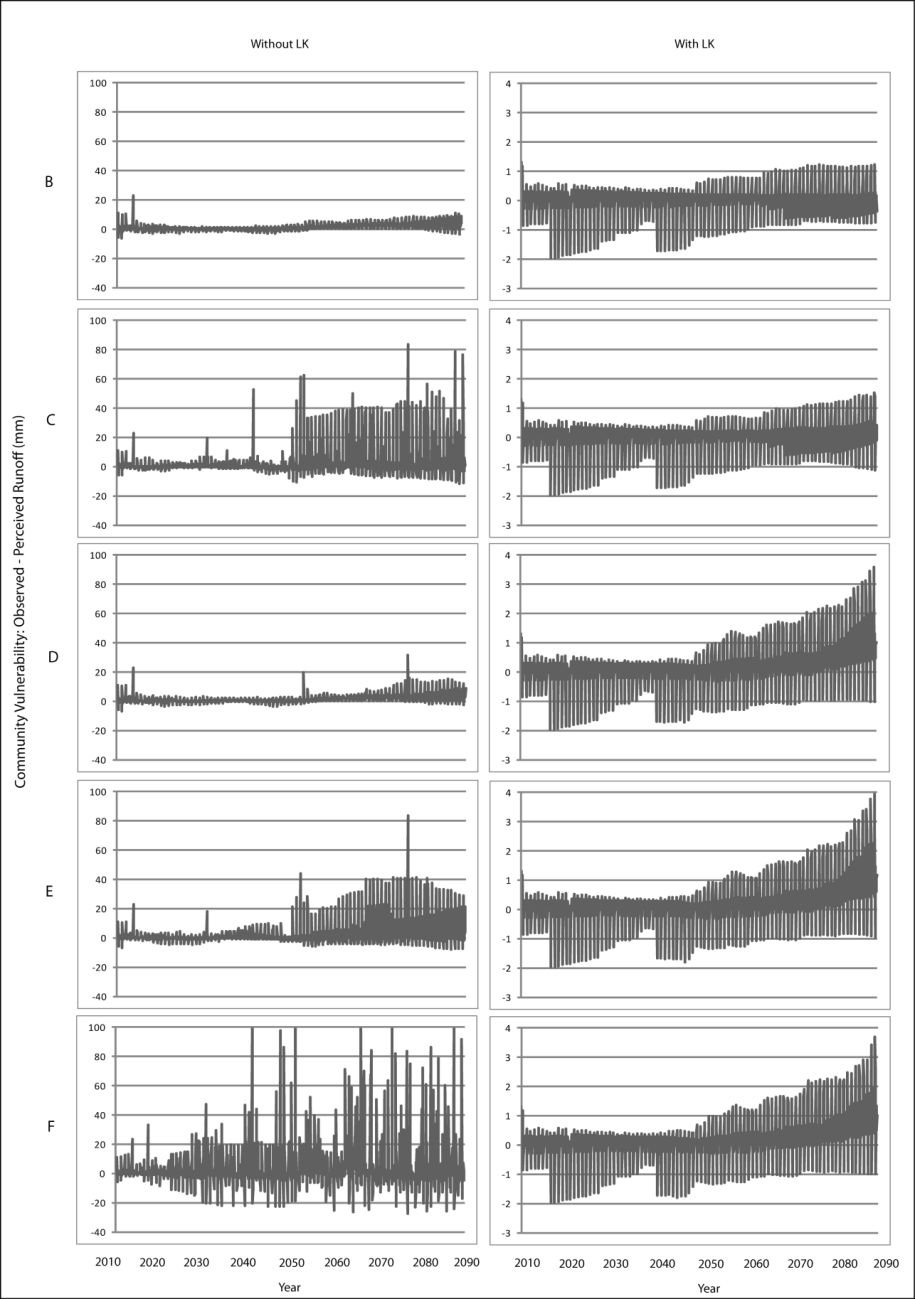
Simulation results from Scenarios B-F depicting vulnerability to runoff for Teller.

Scenario B demonstrates a gradual increase in vulnerability to each climatic variable is presented for Wales (Figures 7–9) and Teller (Figures 10–12). This is similar to Scenario A and can be attributed to the nature of demographics. There is minimal discrepancy between Scenario A and Scenario B, suggesting that a minimal distancing will only have marginal impacts on the ability of communities to accurately perceive changes to environmental variables over time.

Comparing Scenarios C and D, it is apparent that a decline in NMS for the young age class is far more detrimental than a NMS decline for the older age class when knowledge transmission is not included. In fact, Scenario D resembles the result from the communities with perfect knowledge, suggesting that communities may fair better at minimizing vulnerability to climate change by encouraging youth to engage in traditional resource use instead of focusing on ensuring the older individuals maintain their traditional use. This is because, with the transfer of knowledge, environmental knowledge can be retained for much longer in a community if its youth are engaged with traditional resource use that provides opportunity to experience changes in the local climate over time.

The results for Scenario E demonstrate the impacts of a gradual decline in NMS by all agents dependent on age. While this scenario depicts a community with an increase in vulnerability, the results are counterintuitive as the community that exhibits less vulnerability to climate change over time than does the community in Scenario C. This finding emphasizes the impacts that a sharp decline in youth engagement in NMS can have (*i.e.*, Scenario C), and suggests the existence of some threshold at which distancing causes a more serious impact to vulnerability. Such a threshold is passed when all agents in a community have a sharp decline in use of NMS (Scenario F) as the annual variability of vulnerability does not follow a distinguishable pattern, suggesting that a community`s ability to accurately perceive change resembles the pattern for a randomized process.

### 4.3. How Does the Inclusion of LK Influence Community Perceptions and Mitigate Adverse Impacts of TIED on Community Perceptions?

Community perception of climate change significantly improves when LK is incorporated. For Scenario A, agents now have a perfect knowledge of climate change that has occurred over the entire time series. However, community vulnerability is not completely eliminated in this scenario due to the weights used to transfer knowledge between the different types of agents, but vulnerability remains far less than when LK is not included in the simulation. While this observation is intuitive, it demonstrates the model’s ability to adequately simulate the difference in community vulnerability between the use and non-use of LK.

A notable observation with the LK simulations is that changes in community perception appear to occur at specific time steps rather than as a gradual process as is the case when LK is not incorporated. That is, with LK, the trajectory of vulnerability shows sudden shifts. These shifts are moments in time when an older agent from who others seek knowledge dies, which results in the removal of their knowledge of climate change. Although their knowledge is passed onto other agents, the manner in which knowledge is transferred will potentially change.

Converse to the results without LK, Scenario C exhibits less vulnerability than Scenario D, suggesting that it is more important to ensure that, when knowledge transfer is utilized in a community, elders retain their use of NMS in order to aid the ability of the community to perceive change. This observation is intuitive as those communities whose vulnerability rests on the transmission of knowledge from older agents need to ensure that those agents remain engaged with traditional resource use in order to be able to pass along sufficient knowledge to younger agents. What is important to note here is that the inclusion of knowledge transfer can affect a community depending on the extent to which they are engaged with traditional resource use.

### 4.4. How Is Community Perception Influenced by Community Structure?

The two study sites of Wales and Teller exhibit minimal difference in demographics and climate variables. As a result, any noticeable discrepancies between the two sites should be attributed to the community structure as defined by agent types as Teller has a smaller proportion of gamma agents. However, there is no noticeable difference as vulnerability in all simulated scenarios appears to be similar with regards to the general emerging patterns of perceptions. Thus, the presence of a relatively small proportion of gamma agents does not seem to impact vulnerability over the long run. This does not diminish the roles of agent types in determining community perceptions as the lack of differences in the results between the two sites are potentially due to the overriding impact that alpha and beta agents have when affecting community vulnerability. This leads to the question of what proportion of gamma agents will introduce a significantly adverse impact on community perceptions of climate change?

## 5. Discussion

Remote resource-dependent communities provide simple systems for attempting to understand how to adapt to the potential impacts of climate change. A lack of resources and infrastructure along with a small population base provide challenging circumstances from which mitigation from, or adaptation to, vulnerabilities can be developed. It is, therefore, crucial to understand the mechanisms that may lead to an improvement in the ability of communities to cope with change. This study presents an abstract analysis of how knowledge and technology interact to influence the ability of communities to accurately perceive changes occurring in their local climate. The agent-based model was developed to represent the process by which an individual estimates how their climate is changing, how this and all other perceptions in a community are aggregated to form a single estimate of change, and how this aggregated view differs from simulated climate observations. The objective was not to determine *if* agents could accurately perceive change, but instead the nature in which community perceptions vary from observations of climate-driven change given the impacts of TIED and the transmission of LK.

First, it should be expected that vulnerability at some level will occur due to community demographics, as knowledge about the local climate of the past is lost from the community when an older agent, from who others seek knowledge, dies. Furthermore, vulnerability will exist due to the process of knowledge transfer between different individuals. These expectations were verified as the results showed that even when perfect knowledge and knowledge transfer are in place, the fact that communication of knowledge must take place between agents leads to some level of vulnerability. We would also expect that communities that are composed of mostly alpha and beta agents should experience less vulnerability than those with a greater proportion of gamma agents; however, the results showed that a small number of additional gamma agents will likely not impose a significant impact. Future research in this area should examine the existence of thresholds with regards to the number of gamma agents that would eventually cause a significant difference in overall community perception.

Second, determining how to assist communities in adapting to climate change should be based on the collection of behaviours that are exhibited by individuals. For example, it is inappropriate to assume that because a certain age class is or is not engaged in traditional resource use that they should be the focus of efforts to minimize vulnerability. The results demonstrate that without the inclusion of knowledge transfer it is more advantageous to focus on the younger individuals in the community with regards to engaging them in practices that will help them experience and properly assess their local climate. Because these agents will exist for the longest periods of time in the community (excluding the potential for migration), it is important for them to not be overly influenced by modernization of resource use (*i.e.*, TIED), and instead acquire accurate environmental knowledge and contribute to the community’s environmental perceptions. Conversely, when knowledge transfer is incorporated, it is more beneficial to allocate resources to not allowing older agents to lose their time spent engaging in traditional resource use because they are responsible for providing knowledge to younger generations. As a result, younger individuals may still be able to participate to some degree in non-traditional resource use (e.g., municipal water systems), which subsequently may provide for more time to spend on other tasks beneficial to the community, while not completely losing knowledge about the climate that they would otherwise need to gain from engaging in traditional resource use (e.g., non-municipal water systems). Thus, solutions for addressing vulnerability to climate change should be focused on the specific social and demographic structures of communities (e.g., the agent type composition) as it is unlikely that specific plans for building resilience in one community can be readily transferred to others.

Third, when knowledge transfer is utilized, there exists the potential for trajectory shifts in community vulnerability. These shifts, albeit relatively small in the results of this study, are caused by the death of individuals who others look to for knowledge and the replacement by another agent who has a different knowledge of the local climate or who is a different individual type of agent with regards to their concern for the general wellbeing of the community. These trajectory shifts are of little concern when the movement is towards a decline in vulnerability or if the shifts cause little change in the long-term; however, there are instances with the use of LK when the trajectory shifts towards an increase in vulnerability, which is especially notable during the midpoint of the time series (*i.e.*, around 2050) in some scenarios. This finding stresses that it is important to understand the structure of those agents that are in a position to provide knowledge to younger generations. Are all these agents concerned for the well-being of the community? Do these agents have equivalent memories of climatic variations in the past? Thus, while the overall structure of a community is important for transferring knowledge, it is also crucial to understand the behaviours and knowledge of those individuals who will potentially play the role of ‘knowledge provider’.

The study presented here is part of a larger research endeavour that is not only concerned with the vulnerability of small remote communities in Alaska, but also the ability of indigenous communities around the world to be able to adapt to the projected impacts of climate change. The general framework of the model presented here facilitates its use for a variety of case studies in other areas in Alaska as well as other regions in which small communities are vulnerable to climate change due to climate dynamics, demographics, the transfer of knowledge within communities, and presence of TIED. However, the generalized nature of the model does limit the scope of questions that can be addressed. For example, issues regarding the impact that agent migration, the availability of environmental knowledge from outside the community, and specific agent-agent relationships can have on community vulnerability require further data collection and modifications to the model that will allow such parameters to be included. Furthermore, while the manner in which large-scale climatic data was synthesized with social data pertaining to local communities provides insight on how communities are situated to perceive changes in climate, it does present challenges with validation as it is difficult to collect empirical data for evaluating model outcomes of the different scenarios that are hypothetical in nature and exist over relatively long time periods with regards to social data.

Perceptions are only a single component in the complex social-ecological system of community vulnerability to environmental change, but it is one of the main drivers of vulnerability and can be analyzed and addressed in the hopes of building resilience and strengthening adaptation. In order to advance these conclusions the following questions provide a basis for future research: *how does communication between individuals in a community influence the transfer of knowledge?; given a community’s engagement with traditional resource and its use of LK, what mechanisms can be in place to assist specific age groups with minimizing vulnerability?; does a sub-network of ‘knowledge-providers’ exist within a community, and, if so, how does their behaviours and interactions impact the community’s overall perceptions?; what climatic variables are most important for a community in perceiving climate change, and how does the short- and long-term variability of such variables influence community vulnerability?* This sets an agenda for understanding community response to climate change and for maintaining community well-being and health.

## Figures and Tables

**Table 1.** Variables used in agent-based model.

**Model Variables**	**Description**
*M*	Month
*y_c_*	current year
*y_p_*	previous year
*w_k_*	weight knowledge passed between agents of different types
*i_age_*	age of agent *i*
*age class*	Y = younger (18–39 years)
	M = middle (40–59 years)
	O = older (over 60 years)
*t_TRU_*	time engaged in traditional resource use
*X*	climate variable (*i.e.*, precipitation, runoff, temperature)
*p_i,x_*	agent *i*’s perception of change in variable *x*
*j_LK_*	local knowledge of all agents other than agent *i*
*P_c,x_*	community perception of change in variable *x*
*q_x_*	recorded change in variable *x*
*v_x_*	community vulnerability to change in variable *x*

**Table 2.** The weight, *w_p_*, expressing the influence that each agent has on community perception based on agent type and agent age class.

	**Alpha**	**Beta**	**Gamma**
**Younger**	0.6	0.3	0.0
**Middle-aged**	0.8	0.4	0.1
**Older**	1.0	0.8	0.7

**Table 3.** The weight, *w_k_*, expressing the amount of knowledge that is between individuals of different types.

	**Alpha**	**Beta**	**Gamma**
**Alpha**	1.0	0.8	0.5
**Beta**	0.8	0.5	0.3
**Gamma**	0.0	0.0	0.0

**Table 4.** The six scenarios simulated by the agent-based model as defined by the proportion of time an agent is engaged with non-municipal water systems (NMS). The scenarios are (A) *perfect knowledge*, (B) *traditional resource use by all agents*, (C) *diminishing NMS by younger agents*, (D) *diminishing NMS by older agents*, (E) *gradual diminishing of NMS by all agents*, and (F) *rapid diminishing of NMS by all agents*.

	**Younger Age**		**Middle Age**		**Old Age**	

**Scenario**	**Initial**	**Annual Change**	**Initial**	**Annual Change**	**Initial**	**Annual Change**
**A**	1	0.0%	1	0.0%	1	0.0%
**B**	0.95	0.0%	0.95	0.0%	0.95	0.0%
**C**	0.95	–5.0%	0.95	1.0%	0.95	0.0%
**D**	0.95	0.0%	0.95	1.0%	0.95	5.0%
**E**	0.95	–4.0%	0.95	2.5%	0.95	1.0%
**F**	0.95	–15.0%	0.95	10.0%	0.95	5.0%

**Table 5.** The 5-year average of each variable from the start and end of the time series, and the difference between these two dates.

	**1962**	**2088**	**Difference**
**Temperature**	−4.4	3.9	8.3
**Precipitation**	23.9	45.0	21.1
**Runoff**	5.6	13.0	7.4
